# Hair Dyes

**Published:** 1872-12

**Authors:** 


### HAIR DYES

Contain sugar of lead, in most cases, in variable proportions, and often cause lead poisoning, showing itself in a paralysis of the muscles of the fingers, which are not easily straightened; the hand is not readily opened; there is a tendency of the wrist to fail on using it, called "wrist drop;" colicky pains are more or less frequent. Because no red lines are seen on the gums, a uniform symptom of the disease, these affections are liable to be mistaken for some form of rheumatism, and valuable time is lost. The only cure is the discontinuance of the use of the dye. The symptoms will begin to disappear at once, especially if the system is kept open and free, and the diet is made to consist of coarse breads, fruits, oat-meal gruel, and wheaten grits.

